# Whole-Genome Sequencing Reveals Genetic Diversity and Structure of Taiwan Commercial Red-Feathered Country Chickens

**DOI:** 10.3390/ani16020286

**Published:** 2026-01-16

**Authors:** Ya-Wen Hsiao, Kang-Yi Su, Chi-Sheng Chang

**Affiliations:** 1 Genome and Systems Biology Degree Program, Academia Sinica and National Taiwan University, Taipei 10617, Taiwan; d13b51007@ntu.edu.tw (Y.-W.H.); suky@ntu.edu.tw (K.-Y.S.); 2 Department of Animal Science, Chinese Culture University, Taipei 11114, Taiwan

**Keywords:** whole-genome sequencing, genetic diversity, population structure, Taiwan commercial red-feathered country chicken

## Abstract

Whole-genome sequencing is a powerful approach for exploring genomic diversity in livestock species. Chickens (*Gallus gallus*) are an important food source worldwide, and in Taiwan, poultry production contributes substantially to the livestock industry. Taiwan’s commercial red- and black-feathered country chickens dominate this category and play a crucial role in local poultry production. However, fundamental genomic information on their population structure remains limited. To address this gap, this study generated whole-genome sequencing data from red-feathered country chickens originating from four major breeding farms. Genetic diversity analyses revealed uniformly low genetic diversity across all farms. Runs of homozygosity (ROH) analyses indicated predominantly historical inbreeding, with farm-specific differences in recent inbreeding patterns. Population structure analyses revealed clear clustering of individuals according to farm origin, indicating distinct line structures among breeding farms. These results provide the first comprehensive genomic overview of Taiwan’s commercial red-feather country chickens and offer valuable reference information for future breeding strategies and the development of new lines.

## Simple Summary

Red-feathered country chickens are an important food source in Taiwan and are widely appreciated for their meat quality. Understanding their genetic makeup is important because it helps farmers improve breeding programs. In this study, we examined chickens from four major breeding farms in Taiwan. We used whole-genome sequencing to learn how the chickens from different farms are related to one another. Our results showed that chickens formed distinct groups by farm, reflecting each farm’s unique breeding line and practices over time. The findings provide a foundation for future efforts to improve breeding, protect genetic diversity, and support the long-term development of high-quality poultry production in Taiwan.

## 1. Introduction

Chicken (*Gallus gallus*) is one of the most important livestock species globally and plays a particularly significant role in Taiwan’s poultry industry. According to the 2024 Taiwan Poultry Production Statistics Handbook, poultry output reached NT$102,398 million, accounting for 49.40% of total livestock production, and commercial country chickens represent a major component of Taiwan’s domestic poultry production. Among these, Taiwan commercial red-feathered and black-feathered country chickens account for approximately 85% of total country chicken production, highlighting their economic and agricultural importance.

The genetic diversity and population structure of commercial chicken populations reflect their breeding histories and current management practices. Previous studies on Taiwan country chickens have primarily relied on microsatellite markers to examine genetic characteristics [1,2,3]. Although informative, microsatellite-based assessments lack genome-wide resolution. In contrast, the rapid development of whole-genome sequencing (WGS) technologies has enabled more comprehensive investigation of genetic diversity, inbreeding levels, and population stratification in poultry [4,5,6]. For example, WGS analysis of indigenous chicken breeds in Guangxi revealed that their genetic diversity was significantly higher than that of commercial chickens, with clear differentiation among geographically distinct populations [7]. Additionally, a WGS study of the Ningxia Jingyuan chicken successfully identified breed-specific population markers and assessed inbreeding levels using runs of homozygosity (ROH) analysis [8].

Despite these advances, genomic information on Taiwan commercial country chickens remains limited [2,9]. Many commercial populations have undergone long-term self-propagating breeding, which may lead to reduced genetic diversity, uneven population structure, or unintended genetic drift [2]. To date, no study has applied WGS to systematically characterize the genetic diversity, inbreeding status, and population differentiation among Taiwan’s major commercial country chicken lines.

To address this gap, this study performed whole-genome sequencing and population genetic analyses on Taiwan commercial country chickens. The results provide a comprehensive assessment of their current genetic status and offer an important reference for future breeding strategies, conservation planning, and the development of improved commercial lines.

## 2. Materials and Methods

### 2.1. Sample Collection

**Figure 1:**
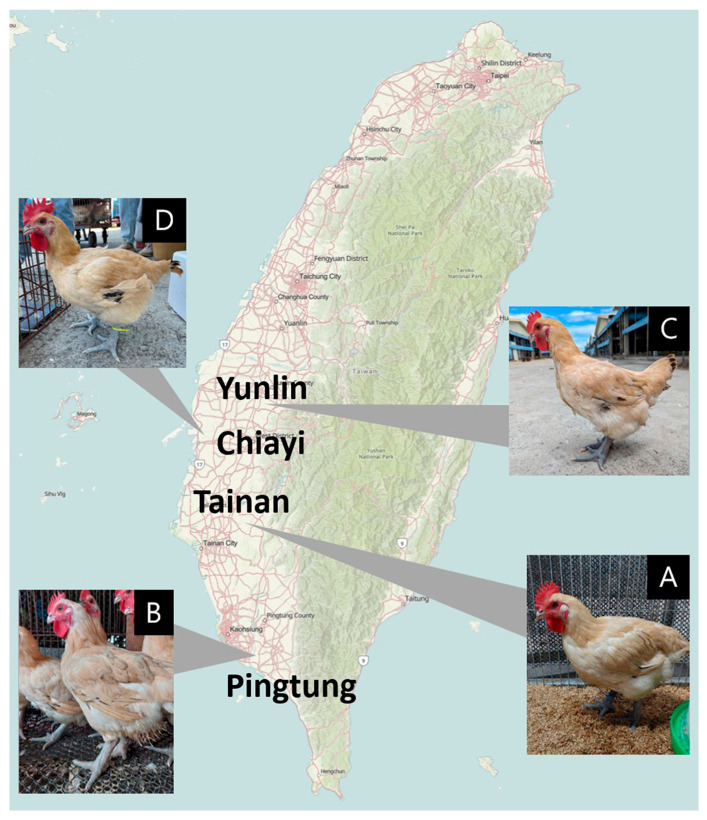
The distribution map and the appearance of the Taiwan commercial red-feathered country chickens in this study. (**A**) refers to the farm located in Tainan County, (**B**) refers to the farm located in Pingtung County, (**C**) refers to the farm located in Yunlin County, and (**D**) refers to the farm located in Chiayi County.

The four major Taiwan commercial red-feather country chicken farms included in this study collectively house about 287,000 hens, representing 35.3% of the national breeding population. Due to the commercial sensitivity of the information, blood samples in this study were collected exclusively from female offspring of parent stock (PS) at eight weeks of age. The four populations were designated as A (*n* = 30), B (*n* = 30), C (*n* = 30), and D (*n* = 30), resulting in a total of 120 individuals (Figure 1). At the same time point, approximately 10% of the female offspring were retained as breeders, while the remaining birds were sold to the market. Detailed information on each breeder farm is in Supplementary Table S1. Blood was drawn from the wing vein of each bird and stored in anticoagulant tubes at −20 °C. Genomic DNA was extracted using the QIAGEN DNA Extraction Kit (QIAGEN, Crawley, UK). DNA quality and concentration were assessed using a NanoDrop ND-2000 spectrophotometer (Thermo Fisher Scientific, Waltham, MA, USA), a Bio-Fragment Analyzer (BiOptic, New Taipei City, Taiwan), and agarose gel electrophoresis. All animal procedures were approved by the Institutional Animal Care and Use Committee (IACUC) of Chinese Culture University (Approval No. CCU-IACUC-113008A3). The study followed the guidelines for the care and use of laboratory animals, and the protocol was valid from January 2024 to December 2026.

### 2.2. Library Preparation and Whole-Genome Sequencing

Genomic libraries were prepared using the Illumina DNA Prep kit (Illumina, San Diego, CA, USA), and library quality was evaluated using the Bio-Fragment Analyzer (BiOptic, New Taipei City, Taiwan). Libraries were pooled in approximately equal proportions and subjected to 650 bp paired-end sequencing on the Illumina NovaSeq X Plus platform (Illumina, San Diego, CA, USA).

### 2.3. Quality Control, Read Alignment, and Variant Calling

Base calling and demultiplexing were performed using bcl2fastq (v2.20.0.422). Raw read quality was assessed using FASTQC (v0.11.9). High-quality paired-end reads were aligned to the *Gallus gallus* reference genome (GRCg7b; GCF_016699485.2) using the DRAGEN Bio-IT Platform (Illumina, San Diego, CA, USA). Reference-based variant calling and depth-based filtering were performed within the DRAGEN pipeline. Variants were further processed using bcftools (v1.23), and genotype data were filtered using PLINK (v1.9). The retained SNPs met the following criteria: at least 85% of samples with depth > 1500 per SNP, MAF > 0.05, missing rate per individual < 0.1, and missing rate per SNP < 0.15 [10]. After filtering, 109,795 SNPs and 12,247 InDels were retained for downstream analysis. Variant annotation was performed using SnpEff (v5.4) [11]. SNP and InDel density distribution plots were generated with the CMplot package in R (v4.3.0) within the RStudio environment (2023.03.1-446) (https://github.com/YinLiLin/CMplot, accessed on 4 July 2024).

### 2.4. Genetic Diversity Analysis

Genetic diversity parameters, including expected heterozygosity (He), observed heterozygosity (Ho), minor allele frequency (MAF), polymorphism information content (PIC), and Nei’s genetic distance, were calculated using PLINK (v1.9) with default settings. The number of effective alleles (Ne) and observed alleles (Na) for each population were estimated using NeESTIMATOR (v2.0.1) [12]. Whole-genome nucleotide diversity (Pi) was computed using VCFtools (v0.1.17) [13].

### 2.5. Runs of Homozygosity (ROH) Analysis

Runs of homozygosity were identified using PLINK (v1.9) to evaluate inbreeding levels and genomic relatedness within and among populations. ROH segments were categorized by length into six classes: 0–0.5 Mb, 0.5–1 Mb, 1–1.5 Mb, 1.5–2 Mb, 2–2.5 Mb, and 2.5–3 Mb.

### 2.6. Principal Component Analysis

Population structure was examined using principal component analysis. Eigenvalues were calculated using the Eigenstrat algorithm implemented in the Golden Helix SNP Variation Suite (Golden Helix, Bozeman, MT, USA). PCA plots were generated using Microsoft Excel (Version 2019, Microsoft Corporation, Redmond, WA, USA).

### 2.7. Phylogenetic Analysis

SNP genotype data were converted to “genind” format using the “gl2gi” function in the dartR package (v2.9.7) within R (v4.3.0) [14]. Nei’s genetic distance was calculated using the “aboot” function in the poppr package (v2.9.4) [15]. A neighbor-joining phylogenetic tree was constructed and visualized using the ape package (v5.8-1) [16].

## 3. Results

### 3.1. Sequencing Quality and Genomic Variants

All samples passed DNA, library, and sequencing quality control (Table 1). The Q20 value of each sample exceeded 95%, and the Q30 value exceeded 90%, indicating high base-calling accuracy. Clean reads were efficiently aligned to the chicken reference genome, with mapping rates ranging from 97.63% to 98.42% across farms. The total sequencing yield per farm ranged from 17.82 to 21.53 Gbp, and the average sequencing depth was consistently within 15.29×–16.40×. Overall, the sequencing quality and depth were sufficient to ensure reliable downstream variant detection and population genomic analyses.

**Figure 2:**
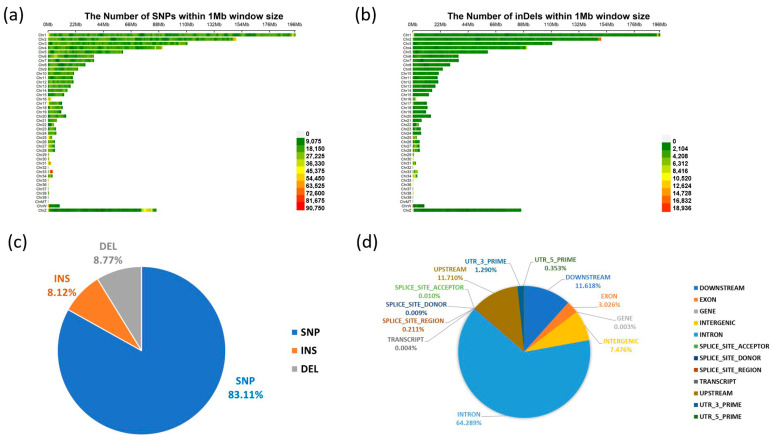
Distribution of SNP and InDel across the whole-genome of Taiwan commercial red-feathered country chickens. (**a**) SNP density statistics across the whole-genome; (**b**) InDel density statistics across the whole-genome; (**c**) Number of variants by type; (**d**) Annotation result for variants.

Across the genome, SNPs and InDels were distributed on all chromosomes (Figure 2). The number of variants generally corresponded to chromosome length (Figure 2a,b). SNPs accounted for 83.11% of all detected variants, insertions for 8.12%, and deletions for 8.77% (Figure 2c). Most variants were located in intronic and intergenic regions. Variant annotations showed the following proportions: intron (64.289%), intergenic (7.476%), downstream (11.618%), upstream (11.710%), 3′UTR (1.290%), 5′UTR (0.353%), exon (3.026%), splice regions (0.230%), and others (Figure 2d). A smaller fraction of variants was found in exonic and splice-site regions, while UTRs accounted for less than 2% of all annotated variants.

### 3.2. Genetic Diversity

Genetic diversity parameters were estimated for each farm (Table 2). The observed number of alleles (Na) ranged from 2.058 to 2.059, and the effective number of alleles (Ne) ranged from 1.386 to 1.407. PIC values varied slightly between 0.177 and 0.179. Observed (Ho) and expected heterozygosity (He) ranged from 0.260 to 0.274 and 0.278 to 0.283, respectively. Minor allele frequency (MAF) showed small variation across farms, indicating similar levels of heterozygosity and allele distribution overall. Farms A and B exhibited slightly higher observed heterozygosity, while Farm C had the highest expected heterozygosity and PIC values. Farm D values were intermediate for most parameters. No statistically significant differences were detected among the farms (ANOVA, *p* > 0.05), suggesting that the four populations maintained relatively uniform genetic diversity.

### 3.3. Runs of Homozygosity

**Figure 3:**
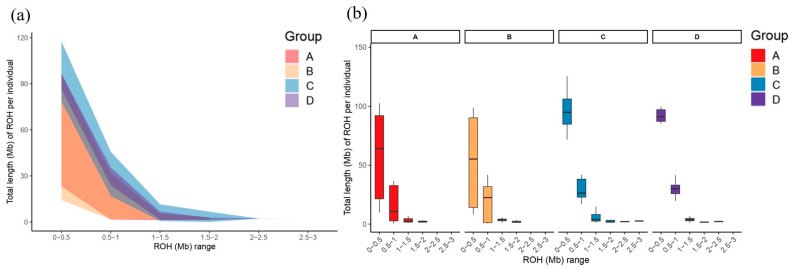
Runs of homozygosity (ROH) profiles in Taiwan commercial red-feather country chickens. (**a**) Genome-wide distribution of ROH segments in Taiwan commercial red-feathered country chickens. (**b**) Total ROH lengths in different ROH length categories (Mb) for each farm. A refers to the farm located in Tainan County, B refers to the farm located in Pingtung County, C refers to the farm located in Yunlin County, and D refers to the farm located in Chiayi County.

Runs of homozygosity (ROH) were classified into six length categories: 0–0.5, 0.5–1, 1–1.5, 1.5–2, 2–2.5, and 2.5–3 Mb (Figure 3). Across all sampled farms, the majority of ROH segments were short, with segments shorter than 1 Mb accounting for 85–90% of the total ROH length. Farm C exhibited the highest total ROH in the 0–0.5 Mb category and was uniquely characterized by the presence of longer ROH segments in the 2.5–3 Mb range, which were absent in farms A, B, and D. Within the 0.5–1 Mb class, farms B and D displayed similar total ROH lengths, whereas farm A showed slightly lower values. Overall, the distribution of ROH lengths indicates that short homozygous segments dominate the genomes across all farms, while longer ROH segments are relatively rare.

### 3.4. Population Structure

**Figure 4:**
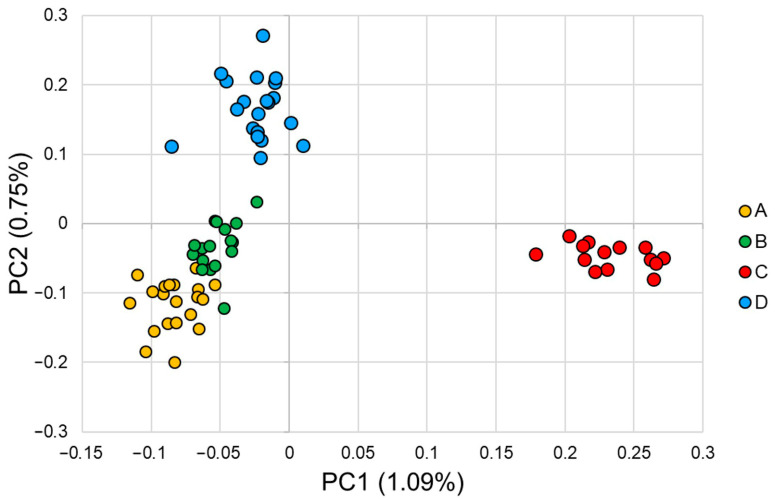
Principal component analysis (PCA) of whole-genome data for Taiwan commercial red-feathered country chickens. Samples are colored by farm (A: yellow, B: green, C: red, D: blue). The *x* axis represents the variance explained by PC1 (1.09%), and the *y* axis represents the variance explained by PC2 (0.75%).

Principal component analysis (PCA) revealed clear genetic differentiation among the four farms (Figure 4). Although the first two principal components accounted for a modest proportion of the total genomic variance (PC1: 1.09%; PC2: 0.75%), they were sufficient to resolve distinct clustering patterns. Individuals from farms A and B overlapped extensively, indicating a high degree of genetic similarity between these two breeder populations. In contrast, samples from farm C formed a well-defined and isolated cluster, suggesting a unique breeding history or limited genetic exchange with the other farms. Farm D exhibited a more dispersed distribution.

**Figure 5:**
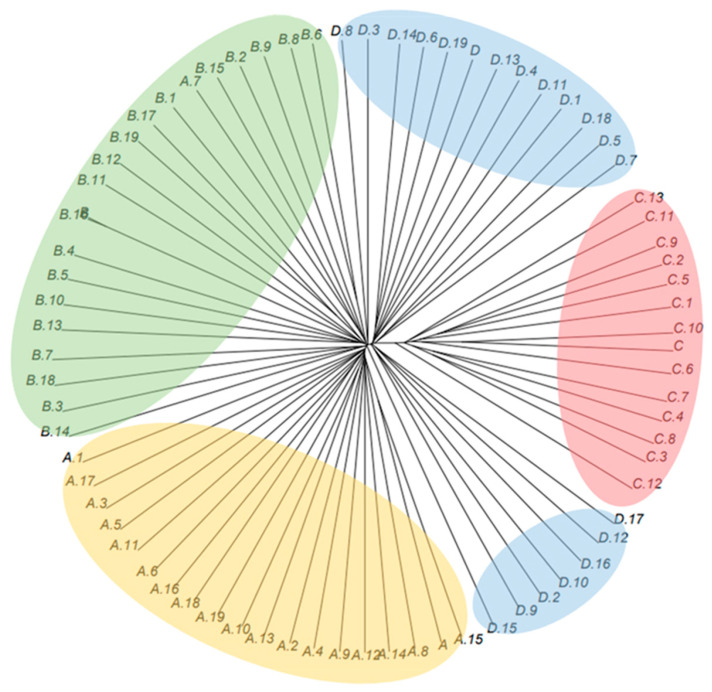
The unroot phylogenetic tree of whole-genome data for Taiwan commercial red-feathered country chickens. Samples are colored by farm (A: yellow, B: green, C: red, D: blue).

The phylogenetic tree based on whole-genome variants was consistent with the PCA results (Figure 5). Individuals formed four clusters corresponding to their respective farms, and the branches separating farms A, B, C, and D were clearly resolved. Farm D was the only population that exhibited two closely related subgroups within its cluster. Samples from farm C were positioned farther from the other farms on the tree, with longer branch lengths indicating greater divergence at the whole-genome level.

## 4. Discussion

The high sequencing quality and consistent depth across all samples ensured robust detection of genome-wide variants in Taiwan commercial red-feathered country chickens. The predominance of variants within intronic and intergenic regions aligns with patterns reported in other chicken genomes, reflecting general genome architecture and the fact that most standing variation resides outside protein-coding exons [17,18]. Recent whole-genome resequencing studies have shown that commercial chicken lines harbor a large proportion of non-coding variation, suggesting strong purifying selection acting on coding regions [17]. Although such variants are less likely to alter protein sequence directly, their potential regulatory impacts—particularly within promoter-proximal or UTR regions—should not be overlooked.

Genetic diversity analyses revealed uniformly low diversity across the four farms. PIC, heterozygosity, and allele-number estimates were lower than those reported for many indigenous chicken breeds [5], and below are previously published microsatellite-based estimates for Taiwan red-feathered chickens [3]. These results highlight the genetic consequences of long-term directional selection, closed breeding structures, and reliance on limited founder stocks—conditions typical of commercial breeding populations. While reduced diversity may reflect successful fixation of desirable production traits, it also raises concerns regarding decreased adaptive potential, reduced robustness under environmental challenges, and increased vulnerability to emerging pathogens. The similarity in diversity measures among farms further suggests that these breeding units are shaped by comparable selection objectives and breeding histories. Such low differentiation among commercial populations has been observed in other studies, where shared founder lines and breeding practices contribute to genomic homogenization [19].

The ROH landscape provides additional insight into historical and recent inbreeding dynamics. The dominance of short ROH across all populations indicates that most homozygous tracts originate from more ancient shared ancestry rather than recent mating among close relatives. This pattern is similar to what has been reported in commercial vs. wild or indigenous chickens, where short ROH segments predominate and reflect longstanding demographic history [19,20,21,22]. However, farm C exhibited both a greater abundance of very short ROH and the unique presence of longer ROH segments (2.5–3 Mb), suggesting a combination of historical relatedness and more recent or farm-specific inbreeding. Longer ROH (>2 Mb) have been interpreted in other chicken studies as evidence of more recent inbreeding events [23]. This pattern may reflect differences in breeding practices—such as smaller effective population size, the reuse of successful sires, or restricted introduction of new genetic material. Although extremely long ROH segments (>5 Mb), which typically signal very recent inbreeding, were absent, the accumulation of intermediate ROH in certain farms warrants ongoing monitoring to prevent future increases in genomic inbreeding.

Population structure analyses consistently revealed moderate differentiation among the farms. Despite explaining little variance, PC1 and PC2 can separate populations due to the high resolution of genome-wide markers (PC1: 1.09%; PC2: 0.75%). A phenomenon similarly observed in other WGS-based studies. For example, in Guizhou indigenous chickens, PCA and phylogenetic analyses delineated clear breed clusters corresponding to geographic and breeding histories (PC1: 4.7%; PC2: 2.5%) [5]. Farms A and B were nearly indistinguishable in PCA space, suggesting shared origins or continued exchange of breeding lines. In contrast, farm C formed a clearly separated cluster, consistent with its unique ROH profile and slightly higher genetic diversity, implying a distinct breeding history or reduced genetic exchange with other farms. The unrooted phylogenetic tree constructed from whole-genome variant data further corroborated the PCA findings. Branch lengths separating farm C from the other populations were relatively long, indicating higher genomic divergence consistent with its elevated ROH-based inbreeding and slightly greater diversity. Farm D exhibited two internal subclusters in the phylogenetic tree, which may reflect the existence of sub-lines, recent admixture, or incomplete homogenization of parental contributions. Overall, while the farms maintain distinguishable genomic signatures, the degree of divergence remains modest, consistent with their shared status as commercial red-feathered chicken breeding populations.

Together, these genomic patterns highlight the importance of active genetic management to sustain long-term population health. Strategies such as increasing the effective population size, rotating or balancing sire and dam contributions, periodically introducing unrelated or minimally related lines, and monitoring ROH-based genomic inbreeding could help mitigate further diversity loss [24,25,26]. Implementing such strategies aligns with recommendations from conservation genomics in livestock, where genomic tools are increasingly used to balance selection gain with inbreeding control. Given the economic and cultural importance of red-feathered country chickens in Taiwan, the genomic information generated in this study provides a valuable foundation for evidence-based breeding programs, supports sustainable genetic improvement, and contributes to the conservation of commercial germplasm.

## 5. Conclusions

This study provides the first whole-genome sequencing dataset and comprehensive genomic characterization of Taiwan commercial red-feathered country chickens. By analyzing genome-wide variants, genetic diversity indices, runs of homozygosity, and population structure, we offer an updated overview of the current genetic status of breeder populations from four major farms in Taiwan. The overall low genetic diversity and distinct clustering patterns among farms highlight the influence of breeding practices and potential differences in breeding histories.

The genomic information generated in this study establishes an essential reference for future breeding, conservation planning, and management of Taiwan commercial red-feathered country chickens. Furthermore, these whole-genome data provide a valuable resource for genome-assisted selection and trait-specific breeding programs, enabling more precise genetic improvement of economically important traits such as growth rate, meat quality, and disease resistance.

## Figures and Tables

**Table 1:** Summary of whole-genome sequencing data for Taiwan commercial red-feathered country chickens.

Farm ^1^	Total Reads	Total Yield (Gbp)	Q30 (%)	Q20 (%)	Mapped Reads	Mapped Reads % (Out of Total Reads)	Mapped Yield (Mbp)	Mapped Mean Depth (×)
A	142,581,649	21.53	94.66%	97.15%	139,883,098	98.09%	21,122.36	16.40
B	132,541,394	20.01	94.79%	97.23%	130,424,493	98.42%	19,694.12	15.53
C	118,019,141	17.82	92.08%	95.50%	115,174,475	97.63%	17,391.35	15.29
D	135,219,896	20.42	94.36%	97.02%	132,407,501	97.92%	19,993.53	15.87

^1^ A refers to the farm located in Tainan County, B refers to the farm located in Pingtung County, C refers to the farm located in Yunlin County, and D refers to the farm located in Chiayi County.

**Table 2:** Whole-genome sequencing revealed genetic diversity in Taiwan commercial red-feathered country chickens.

Farm ^1^	Na ^2^	Ne ^3^	Pi ^4^	PIC ^5^	MAF ^6^	Nei ^7^	Ho ^8^	He ^9^
A	2.058	1.400	0.280	0.177	0.170	0.280	0.274	0.279
B	2.059	1.407	0.279	0.177	0.173	0.279	0.273	0.278
C	2.058	1.386	0.284	0.179	0.164	0.284	0.260	0.283
D	2.058	1.405	0.280	0.177	0.172	0.280	0.273	0.279

^1^ A refers to the farm located in Tainan County, B refers to the farm located in Pingtung County, C refers to the farm located in Yunlin County, and D refers to the farm located in Chiayi County. ^2^ Na: Observed number of alleles. ^3^ Ne: Effective number of alleles. ^4^ Pi: Nucleotide variability. ^5^ PIC: Polymorphism information content values. ^6^ MAF: Minor allele frequency. ^7^ Nei: Nei’s genetic distance. ^8^ Ho: Observed heterozygosity. ^9^ He: Expected heterozygosity.

## Data Availability

The data presented in this study are available on request from the corresponding author.

## Supplementary Materials

The following supporting information can be downloaded at: https://www.mdpi.com/article/10.3390/ani16020286/s1, Table S1: Survey results of four Taiwan commercial red-feathered country chicken farms.
