# Supplementary material for: Whole-Genome Sequencing Reveals Genetic Diversity and Structure of Taiwan Commercial Red-Feathered Country Chickens
Source: Animals (Basel). 2026 Jan 16;16(2):286. doi: 10.3390/ani16020286 (PMC12838120; doi:10.3390/ani16020286)
Supplement: Supplementary file 1 [file animals-16-00286-s001.zip › animals-4032778-supplementary.pdf]

Supplementary Table S1. Survey results of four Taiwan commercial red-feathered country chicken farms.

**Farm name**	**A**	**B**	**C**	**D**
**Type**	Main breeder farm	Main breeder farm<br>Others 5	Main breeder farm<br>Others 4~5	Main breeder farm<br>Others 4~5
**Number of chickens**	Male 1,000<br>Female 60,000	Female 115,000	Female 62,000	Male 1,000<br>Female 50,000
**Hatch by own**	Yes	Yes	No	No
**Number of chickens hatched per week**	80,000	120,000	80,000	50,000
**Breeder chicken source farm**	Own	Own and cooperate	Own	Own
**Distribution areas of broiler chicken farms**	Taichung	Chiayi	Changhua to Tainan	Chiayi, Yunlin
**Breeder selection weeks**	Male 11 weeks<br>Female 8 weeks	Male 10/11 weeks<br>Female 8 weeks	Male 10 weeks<br>Female 8 weeks	Male 10 weeks<br>Female 9 weeks
**Number of weeks caged for breeder chickens**	Male 11 weeks<br>Female 8 weeks	Male 10/11 weeks<br>Female 8 weeks	12 weeks	9-10 weeks
**Chicken appearance photos**	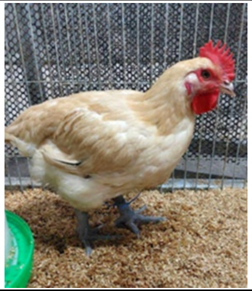	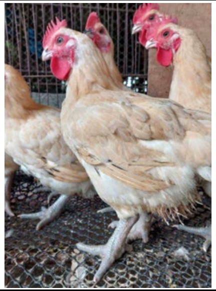	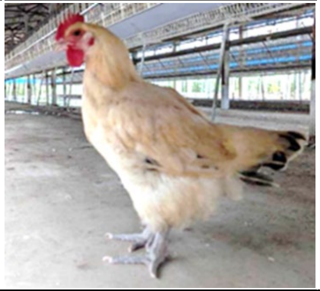	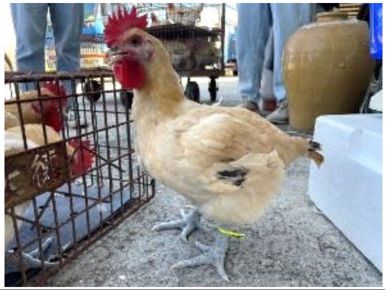
